# Construction at Work: Multiple Identities Scaffold Professional Identity Development in Academia

**DOI:** 10.3389/fpsyg.2019.00628

**Published:** 2019-03-26

**Authors:** Sarah V. Bentley, Kim Peters, S. Alexander Haslam, Katharine H. Greenaway

**Affiliations:** ^1^School of Psychology, The University of Queensland, Brisbane, QLD, Australia; ^2^Melbourne School of Psychological Sciences, The University of Melbourne, Melbourne, VIC, Australia

**Keywords:** social identity, identity construction, professions, academia, education

## Abstract

Identity construction – the process of creating and building a new future self – is an integral part of a person’s professional career development. However, at present we have little understanding of the psychological mechanisms that underpin this process. Likewise, we have little understanding of the barriers that obstruct it, and which thus may contribute to inequality in career outcomes. Using a social identity lens, and particularly the Social Identity Model of Identity Change (SIMIC), we explore the process of academic identity construction among doctoral students. Through thematic analysis of semi-structured interviews with 22 Ph.D. candidates, we observe that the identity construction process relies on a person’s perception of a navigable pathway between their current self and their future self. Importantly, participants who were able to access multiple identity resources were more likely to perceive a navigable pathway to a future professional self (e.g., as an academic), unless they perceived these identities to be incompatible with those held by leading members of the profession (e.g., their supervisors). This research suggests that the identities that people are able to access as they progress in their careers may play an important role in their ongoing professional identity construction and career success.

## Introduction

Seeing a successful woman that still has her identity as part of her persona in research. It’s something that I haven’t seen, and it frightens me. Frightens me to be two persons. That’s not the way it should be.

Female Ph.D. candidate, Engineering

Career pathways can be seen as a progression of role changes: who one was in the past; who one is now; and most importantly, who one will become in the future (Obodaru, 2012). From this perspective, it is clear that careers are characterized by the ongoing relinquishment of old identities (who one was) and construction of new ones (who one is going to be). This process of identity construction is far from simple because it not only involves the acquisition of observable knowledge and skills (the “content” of a job role), but also the internalization of a range of (often implicit) behaviors, values and understandings that are embedded within the future identity (Katz and Kahn, 1978; Ibarra, 1999; Dukerich, 2001). For this reason, this process of future self-construction may have less to do with pragmatic “doing” concerns, and more to do with an experiential sense of who one *wants to be*, or more importantly, *who one thinks one is able to become* (Ibarra and Barbulescu, 2010).

There is evidence that people’s ability to construct a future professional identity matters. Specifically, people who have a clear sense of their future professional identity report higher levels of career motivation and proactive career behavior (Markus and Nurius, 1986; Ibarra, 1999). All else being equal, then, people’s professional identity construction is likely to be an important factor in their career outcomes. And by implication, barriers to professional identity construction may account for unequal career outcomes. The quote above from a female engineering candidate vividly illustrates a break-down in the identity construction process, as she describes the seemingly irreconcilable nature of who she thinks she is and who she believes she must become in order to succeed. In particular, she claims that if she is to succeed she needs to shed her identity as a woman. Understanding how these fissures contribute to what has become known as the leaky pipeline (White, 2004; Clark Blickenstaff, 2005) may provide a new dimension to understanding workplace inequality.

In this paper, we aim to explore people’s experiences of professional identity construction in order to better understand the factors that may facilitate or hinder it. We focus particularly on the importance of multiple social identities in the identity construction process. There are two reasons for this. First, a growing body of work on the social identity model of identity change (SIMIC; Iyer et al., 2009; Jetten and Pachana, 2012) has shown that people who are able to access multiple social identities are better able to cope with a range of life transitions. We ask whether the same social identity processes that underpin lived experiences of identity transition also underpin the yet-to-be-lived process of future identity construction. Second, there is evidence that people’s perceptions of incompatibility between their social identities (e.g., relating to gender or race) and those that characterize prototypical members of the profession can reduce their sense that the career path is feasible and undermine their motivation to pursue it (e.g., Cheryan and Plaut, 2010; Peters et al., 2015). We will now review the literature that speaks to the role of social identity in identity change and its potential relevance to identity construction.

### Multiple Identities and Identity Construction

The evolution of a person’s career is a process that involves identity transition, whether in the course of the educational journey, job promotion, or in the process of moving from one professional domain to another (noting that the average person changes career five times in their life time; Barrett, 2017). Each time a person transitions from one role to another this requires a re-construction of the self – I was a waiter but I am now a restaurant manager, I was a nurse but I am now a doctor, I was a student but I am now an academic. So how does this identity construction process happen, and what are the barriers to its success?

In exploring questions of identity in the workplace, researchers have taken a wide range of approaches, from examining the sense of self a person derives from who they are as an individual with unique capabilities, to that which they derive from their relationships at work or their membership of some larger collective, like a work team or organization (see Miscenko and Day, 2016, for a detailed review). However, regardless of the level at which the identity has been construed, the majority of this work has focused on static identity processes rather than dynamic ones associated with identity change, transition and construction (Ashforth et al., 2008). There are some exceptions to this rule, including some work on relational identification (Sluss and Ashforth, 2008), role modeling (Kelman, 1961) and possible identities (e.g., Ibarra, 1999). Although these exceptions have generated some useful insights, in this paper we take a different approach. In particular, we focus specifically on the role of people’s multiple social identities in identity construction because a growing body of work has revealed that how a person responds to change is affected by their ability to access these social identity resources.

This work into the SIMIC (Iyer et al., 2009) was developed out of the social identity perspective. Originating from the work of Henri Tajfel, this perspective describes how a person’s sense of themselves – their self-concept – can be informed and strengthened through identifications with others (Tajfel and Turner, 1979), and argues that this process of identification is structured through a sense of shared group membership. Self-categorization theory, a socio-cognitive account of the mechanics of social identity salience and social identification, describes how a person’s selfhood is in fact continually constructed and reconstructed according to the context in which they find themselves (Turner et al., 1987; Oakes and Haslam, 1994). These constructions of the self-occur as people navigate their daily lives. For example, a mother dropping her child at kindergarten will likely define herself differently in that instance to the way that she defines herself 30 min later when she enters her law office to begin her day’s work. These changing self-definitions have a direct impact on how people think, feel and behave (Turner, 1982; Turner et al., 1994). In the years since Tajfel’s and Turner’s initial exposition of social identity theory, a wealth of research has explored not only the contextual mechanics of identification, but also the importance of positive identification for healthy functioning and motivation in a broad range of domains (Jolanda et al., 2009; Jetten et al., 2012, 2015, 2017; Greenaway et al., 2015; Bentley et al., 2017; Haslam et al., 2018).

But of particular importance for identity construction, social identity research has been extended to the SIMIC model to speak to the way in which the groups that people belong to can also predict positive outcomes when undergoing identity transitions (Haslam et al., 2008; Haslam and Ellemers, 2011; Best et al., 2014; Dingle et al., 2015). The SIMIC describes how people who are able to access more identity resources in the form of multiple social groups are better able to navigate identity changing events like parenthood, education, illness, or retirement (Iyer et al., 2009; Jetten and Pachana, 2012; Steffens et al., 2016; Tabuteau-Harrison et al., 2016; Praharso et al., 2017; Ng et al., 2018). Two reasons have been advanced for why having multiple identities can facilitate the change process (Jetten et al., 2009). The first is that having multiple identities increases the likelihood that some identities will be maintained over the course of the change, thereby providing a sense of *identity continuity*. The second is that having multiple identities – by virtue of providing people with a lived experience of the rich dimensionality of one’s sense of self – provides a stronger and broader platform for the development of *new social identities*.

Although the phenomenon of identity construction differs from the kinds of transitions that the SIMIC has thus far been applied to (i.e., a lived experience of identity change, such as parenthood, retirement or illness, rather than a yet to-be-lived identity change possibility) there is reason for thinking that it may matter for identity construction too. In particular, if multiple groups can facilitate a sense of continuity from the past to the present, they may also act as a bridge to the future. And if these groups help people to develop new identities they may also facilitate the identity construction process. For instance, it may be that people’s ability to access multiple identities (e.g., as a scientist, supervisor, mentor, analyst, team member) in the context of their profession – both in terms of the number of groups that they belong to and the number of ways they can understand who they are and what they do – affects the ease with which they are able to construct a future identity (e.g., as organizational leader). However, as we discuss next, it is possible that not all identities are equally able to facilitate identity construction.

### Barriers to Identity Construction

The SIMIC argues that people are better able to successfully navigate change if they have multiple identity resources (providing a person with access to a range of support and self-definitions) and these provide some bridging continuity into the future (Haslam et al., 2019). Importantly, bridging continuity can relate to the compatibility between pre-transition and post-transition identities, which can manifest in terms of perceived similarity. As suggested in the quote at the beginning of this piece, one important basis for this perceived similarity and continuity are demographic characteristics like ethnicity, gender, politics, cultural background, or shared values (Platow and van Knippenberg, 2001; Steffens et al., 2013). That is, it was this students’ tendency to see herself as a woman that interfered with her ability to see herself as getting ahead in her profession because she believed that the women who had achieved this professional outcome had only done so by shedding her gender identity. Further evidence for this possibility comes from research demonstrating how people’s social identities play an important role in their role modeling of leading members of a profession (Morgenroth et al., 2015). For instance, there is evidence that women who identify highly with their gender are particularly likely to have female role models (Lockwood, 2006) and that people generally find high-achieving “elite” individuals, with whom they share few identities, demotivating (Hoyt, 2013).

Data would suggest that inequalities – or perceived incompatibilities – amongst socio-demographic group categorizations are still significantly impacting professional outcomes. And today, researchers continue to struggle when tackling issues of minority group access to majority group roles, such as a lack of women in STEM subjects, or a dearth of female CEOs within the tech industries (Wright et al., 1997; Oakley, 2000; Bosma et al., 2011; Cheryan et al., 2011; Stout et al., 2011). This suggests that we have yet to locate all the leaks in the career pipeline. An understanding of the psychological underpinnings of the identity construction process (and in particular, the role of multiple, compatible identities) may shed light on these early fissures and suggest new interventions for addressing continued unequal outcomes.

### The Study Context

We explore the role of multiple identities in identity construction among Ph.D. candidates. The pinnacle of the educational journey, the receipt of a Doctor of Philosophy degree represents the last stages in educational achievement, in which a candidate works with one or two principal advisors to create a body of work that represents their own significant and novel contribution to knowledge. Looking at identity construction within the academic post-doctoral process provides an ideal domain from which to explore these questions. In a relatively short period of time Ph.D. candidates are required to shift their identity from student to academic, from apprentice to master, and ultimately to construct their own bespoke academic identity upon which their future career depends. And this stage in the education journey is one of the least structured, relying almost exclusively on the relationship between the candidate and their advisory team.

To add further complexity to the process, in recent years, the nature of the doctoral training process has become somewhat contentious. An exponential increase in numbers of candidates being awarded Ph.D.s, and a decrease in the number of academic positions for which they trained means that the process of identity construction for Ph.D. candidates has become increasingly problematic (Larson et al., 2014). And while the increasing globalization and diversity of the student body is arguably a sign that more options have become available to more students, the lack of opportunities suggests that in reality academic career pathways are becoming more competitive, less certain, and ultimately *less* available (Warner and Palfreyman, 2001; Hermans and Dimaggio, 2007; Banks, 2008). For instance, while the last two decades have seen an exponential growth in Ph.D.s being awarded globally, in some countries as few as 5% of Ph.D. recipients will progress into a career in academia (Mangematin, 2000; Economist, 2010). Troublingly, there is evidence that women and other underrepresented groups are less likely to negotiate this transition than the members of dominant groups. Anders (2004) for instance, demonstrates how women self-select out of professional academic pathways due to concerns over their ability to successfully manage both academic and parenting identities. And Hill et al. (1999) discuss how identity disconnections within the mentoring process can be a significant contributing factor when it comes to the *lack* of ethnic minorities in academia.

Together, these factors mean that issues of identity construction – who do you want to be? – as well as identity uncertainty – but who can you really become? (Warner and Palfreyman, 2001; Banks, 2008) are likely to be highly salient for Ph.D. students. In line with the rationale set out above, in this research we investigated the mechanisms of professional identity construction within the Ph.D. training process through the lens of the SIMIC model. Given our interest in the identity construction process, and particularly how a professional identity can be constructed or obstructed, we focused on a demographically varied group of students, and especially those who were underrepresented in terms of gender, age, ethnicity, and stage of candidature. We used qualitative thematic analysis of semi-structured interview data to explore candidates’ narratives of identity and identity construction.

## Materials and Methods

### Sample

Twenty-two participants (2 men, 20 women) were interviewed, all individually. This data was collected as part of a larger study into Ph.D. experiences that also included interviews with Ph.D. supervisors (*n* = 34). Our present analysis focused only on data from students because, having successfully navigated the academic career path, supervisors are poorly positioned to provide insight into identity construction failure. Participants were recruited from a range of disciplines at the same Australian university via the university’s monthly e-newsletter which is distributed to all students. The sample was diverse in terms of discipline and stage of study, but included a disproportionate number of women (91% compared to 52% of the Ph.D. population at the university). Purposive diversity sampling was used in the second round of recruitment in an attempt to achieve a more balanced gender distribution but with very limited success. There are likely to be a range of reasons for this (many of which are germane to the issues that the research was exploring), and this is an issue we return to in the Discussion. The study received ethical approval from the university’s School of Psychology ethics committee.

### Procedure

The semi-structured interview questions were devised by the research team to explore Ph.D. experiences broadly, and were informed by the literature on role modeling and social identity in social and organizational contexts (e.g., see Ellemers et al., 1999; Haslam and Ellemers, 2011). Interview questions were piloted by volunteer students in the department in order to hone the questions and to provide an opportunity for the research team to practice conducting interviews (see Appendix). Interviews were undertaken by four research students under the direction of the first two authors, and were conducted at the university in various meeting rooms, according to the location of the interviewee. The interviewers received extensive training and feedback with the interview protocol before they conducted their interviews and the authors conducted quality control throughout. We did not detect any substantive differences in the kind of data that were generated by the interviews on the basis of our reading of the transcripts and listening to the audio-recordings. Interviews lasted on average 50 min and were recorded, and then transcribed. The study was described to potential interviewees as an investigation into the Ph.D. student–supervisor relationship.

Amongst other things, the interview schedule was designed to elicit (a) interviewees’ perspectives on their own identities in the context of their Ph.D., (b) their thoughts on the proto-typicality of their chosen identity within the academic context, (c) the compatibility of that identity with other identities, and (d) their supervisor’s identity.

Interviews were transcribed using indicators for conversational tempo and emphasis. To increase the clarity of our exposition, we omit these indicators from the quotes presented in text, but they are available to the interested reader in the supplement. Our analysis followed the guidelines for thematic analysis detailed by Braun and Clarke (2006). First, the transcribed interviews were read by the first and second authors, who used a deductive perspective to identify themes that related to identity construction. The second wave of analysis deployed an inductive stance to investigate factors related to the processes under investigation in a more exploratory way. This involved close re-readings of the transcripts and attempts to capture all relevant themes. Once complete, the authors collaborated to distil the initial large thematic output into fitting categories or “headlines” (e.g., as recommended by Braun and Clarke, 2013; Haslam and McGarty, 2014). Once this stage was complete, the transcripts were re-read to ensure the summary results that emerged from this process were faithful to the original data.

## Results

### Sample Overview

The 22 participants came from a broad cross-section of disciplines, ranging from engineering to public health to anthropology, and covered all stages of the Ph.D. process from Year 1 through to Year 3^1^. In Australia, Ph.D. students have minimal coursework requirements, and are expected to complete their Ph.D. within 3 years. The vast majority (91%) of interviewees were women, and 50% were domestic students. Their supervisory teams ranged in number from 1 to 4 members, and the preponderance of supervisors were male. Full descriptive data are presented in Table 1.

**Table 1:** Sample characteristics.

Interviewee	Discipline	Ph.D. year	Gender	Domestic/ International	Sup. team
1	Engineering	2	F	I	M, M, M
2	Speech Pathology	2	F	D	F
3	Human Geography	3	F	I	M, F, M
4	Fisheries	2	F	D	M, M, M
5	Environmental Management	3	F	D	M, M, M
6	Public health	3	F	I	F, M, M
7	Communication	3	F	I	F, M
8	Pharmacy	2	F	D	F, M, F, F
9	Civil engineering	1	M	I	M
10	Civil engineering	1	M	I	M, M, M, M
11	Chemical engineering	2	F	D	M, M, M
12	Physiotherapy	3	F	I	F, M
13	Chemical engineering	1	F	D	F, M, M
14	Occupational therapy	2	F	D	F, F
15	Medicine	1	F	I	M, M, M
16	Education	3	F	D	M, ?
17	Humanities and social science	3	F	I	F, F, M
18	Anthropology	2	F	D	M, M, M
19	Physics	3	F	D	M, F, M
20	Engineering	1	F	I	M, F, F
21	Medicine	2	F	D	F, F
22	Veterinary Science	3	F	D	M, F, F

### Ph.D. Students’ Identity Resources

We observed a great deal of variation in candidates’ responses to the questions of how they described their profession to others, and what other terms were appropriate for describing what they do. This variation is presented in Table 2, along with candidates’ responses to the question of whether they considered their Ph.D. to be an apprenticeship or a job. Generally, we found that the Ph.D. candidates could be distinguished according to whether or not they were able to access multiple identities, and whether these identities related to academia (e.g., student, researcher, scientist) or other vocations (e.g., engineer, clinician, physiotherapist). Importantly, there was evidence that multiple identities – especially when these mapped onto multiple domains – were associated with a clearer sense that the Ph.D. provided a pathway to a future profession. As we will discuss in more detail below, Ph.D. candidates who relied on a single academic identity expressed more confusion and frustration about who they were and where they were headed than candidates who reported multiple, mixed identities. Revealingly, the former group of candidates were more likely to consider their Ph.D. to be an apprenticeship, which is both more limited (with one professional outcome) and less agentic (serving under a master) than a job.

**Table 2:** Identity availability, content and Ph.D. status as job or apprenticeship.

Identity availability	*“What do you call yourself when meeting new people…”*	*“Is your Ph.D. a job or an apprenticeship?”*
Single student identity	Student	*Apprenticeship*
	Ph.D. student	*Apprenticeship in a psychological sense, but job in a regimen sense*
	Ph.D. student	*Both apprenticeship in terms of learning about publishing, but a job also*
	Student	*It’s a provisional driving license*
Single career identity	Marine scientist	*Job and research question*
Multiple academic identities	Statistician, data scientist, data analyst	*Learning and training*
	Student, numerical researcher	*Apprenticeship*
	Researcher, academic, Ph.D. student	*Job, apprenticeship and answering research question*
	Scientist, Ph.D. student	*Answering a specific research question… becoming a master of something not just an apprentice*
	Student, research scholar	*Job*
	Ph.D. student, academic researcher, hybrid career	*More than a job because I am so invested in the outcome*
Multiple academic and career identities	Speech pathologist, Ph.D. student, researcher	*You do kind of have to think of a Ph.D. as a job*
	Pharmacist, Health economist	*Apprenticeship (to become health economist)*
	Civil engineer, lecturer, Ph.D. researcher	*Job*
	Ph.D. student, scientist, physicist	*Job*
	Clinician, researcher, tutor	*Job*
	Occupational therapist, Ph.D. student	*I see it as a job*
	Teacher, student, academic, researcher	*Unpaid job (in order to get to an academic career)*
	Research manager, anthropologist	*Job*
	Ph.D. student, lecturer, engineer	*Job*
	Physiotherapist, Ph.D. researcher	*Job*
	Student, marine biologist	*Job*

#### Single Identities

For those candidates who only reported a single Ph.D. student identity, there was often frustration associated with that identity category. This was articulated in terms of the inadequacy of the title “Ph.D. Student” in representing the day-to-day reality of the role, whether in terms of seniority or job clarity. For example, I11 [F, Chemical engineering], described the unsatisfactory nature of the very word *student* when trying to describe her role:

I11: *Actually, not really because I can’t find a really good expression – because you know, when I say Ph.D. student, student means a person who has courses but I’m a … research student. It seems that there is a, there is a lack of word or expression. I can’t say Ph.D. I can’t say I’m an RHD student because most of the people, they don’t know RHD and RHD doesn’t have a very prestigious weighting. On the other hand, um student it means, it seems to me undergraduate. So no, I think it’s not the best way.*

While in most cases single identities fell in the academic domain, there was one student whose only identity was vocational. Speaking to the particular challenges of a single identity as Ph.D. student, this candidate did not express the same level of identity frustration. For her, the Ph.D. process provided a direct pathway to her future self:

I04: *I think to get where I want to be career wise I can’t not have a Ph.D., but I also think it’s just a good experience to do that whole project and have that ownership of the project as well.*

#### Multiple Identities

Many candidates reported having multiple identities. Some referred to themselves using a variety of academic identities, and others used a mixture of academic identities as well as applied vocational identities. The articulation of multiple identities often manifested in candidates’ discussions of how they dealt with the uncertainties of being a Ph.D. student, and they tended to describe how these multiple identity resources buffered them against the difficulties associated with this uncertainty. A female Ph.D. candidate working in Speech Pathology described her own contextual understanding of her identities in this way:

I02: *Like if I am meeting someone that I haven’t seen them for a quite some time, and if they ask me what I am doing these days, then I would say I am working clinically part time and also doing some post graduate study … and then the conversation would kind of progress.*

Ph.D. candidates who had access to multiple identities from academic and vocational domains had a much stronger and more positive sense of how their Ph.D. identity was contributing to their chosen career pathway. These candidates generally reported on the functional and complementary nature of the Ph.D. identity. A female medical student, for instance, who came into her Ph.D. after a period of working in an applied physiotherapy setting, described how the inclusion of a Ph.D. identity had served to strengthen her original therapist identity:

I21: *I don’t think my Ph.D. has like interrupted that at all and in fact I think it strengthened me in my identity as a physio because it’s enabled me to engage like directly with the core of the profession, which is like finding out more and problem solving and doing, and having an impact.*

However, even for Ph.D. candidates whose multiple identities were all in the academic domain there was evidence of more personal agency, as well as a clearer sense of professional career development. This is revealed by the fact that five out of the six candidates who defined themselves with multiple academic identities described their Ph.D. as a job, as did ten of the eleven who defined themselves as having multiple mixed identities. This conceptualization of the Ph.D. process was associated with a sense that the Ph.D. served an intrinsic purpose and was accompanied by high levels of Ph.D. and career motivation.

I20 [F, Engineering]: *I think, even before I started, I had in mind that it’s like this kind of work, it’s my job. It’s not just showing up to lectures, doing assignments, it’s like my work. It’s 8 till 5 and or more (laughs), it’s my responsibility so before I even started I knew that it was going to be like a job for me.*

#### Scaffolding Identity Construction

The analysis above suggests that SIMIC, and its claim that multiple identities are an important resource in times of identity change, may shed light on processes of identity construction. Specifically, as candidates were able to bring more identities to bare on the task of understanding their Ph.D. they were better able to articulate not only who they were, but also who their Ph.D. would help them to become in the future (see Table 3). We identified two main ways in which multiple identities were able to scaffold the construction of a sense of self in the present and into the future: identity management and identity certainty. The former captures the observation that students who had multiple academic identities could draw on them in creative ways in order to deal with the uncertainty associated with the Ph.D. identity and present themselves in positive and readily understandable ways to others. The latter captures the observation that Ph.D. candidates who had multiple mixed identities manifested much higher levels of certainty about who they were are were going to be; they also were more likely to identify the complementary and mutually reinforcing nature of their multiple identities as they looked to the future. In contrast, students who only were able to draw on a single student identity expressed high levels uncertainty and difficulties in negotiating discussions with others that related to their Ph.D. and in understanding what their Ph.D. was for.

**Table 3:** Illustrative quotes relating to the outcomes of identity resources.

Illustrative quotes (“What do you call yourself”)
**Theme 1: *Identity uncertainty***
Quote 1: Well I can’t find anything but, uhh as you ask right now, something look like, uh Ph.D. student who (.) actually RHD student should say we are research (pause) scholar maybe…yeah maybe. I’m not sure!
Quote 2: I just say Ph.D. student…Actually, I’m a registered doctor in China, but here not a lot of people know that and I try not to tell them…yeah, but here I won’t show that identity because it’s not recognized here… So yeah. I cannot figure out I have a lot of identities yet. So maybe Ph.D. is my only identity. I don’t want this. I want to find out answers. So, it’s quite overwhelming.
**Theme 2: *Identity management***
Quote 1: Um, I think it really depends on the situation. (long pause). On legal matters, uh (pause) I would say that I’m a researcher. And (.) like daily life interaction I would say that I’m a student.
Quote 3: I prefer Marine Biologist in that sense, just because of the connotation that tends to come along with student.
Quote 3: … But I tend to favor scientist because it’s a broad term that people understand you know. You say to someone, particularly in normal populous, you say researcher and they go, “oh what do you do, what is that?” You say a scientist and they automatically think lab coats and stuff.
**Theme 2: *Identity certainty***
Quote 1: I’m a mining engineer soon to be lecturer and professor
Quote 2: I normally tell them I’m doing a Ph.D. That I am doing research into bamboo structural element and also with relatable fire safety, and yeah it’s basically what I say…Depending on the situation, I could also say that I am a civil engineer or I could also say that I am a lecturer.
Quote 3: I usually describe myself as a research manager but also as an anthropologist. And so – because to me they (pause) make us stronger. You know I share knowledge across that boundary…

### Professional Models as Bridges From Present to Future Self

According to SIMIC, one reason that multiple identity resources can help people to cope with identity change is that they can provide continuity that connects who one was, who one is now, and who one will be in the future. Research into people’s connections with those who act as models for a profession suggests that these individuals, by providing a concrete representation of a future professional identity, can also contribute to a sense of continuity between present and future selves (e.g., Ibarra, 1999; Morgenroth et al., 2015; Peters et al., 2015). In this section, we report analysis that explored whether there was any connection between Ph.D. candidates’ ability to access multiple identity resources and their connections with salient professional models (i.e., their Ph.D. supervisors). We make two main observations: (1) candidates with multiple identity resources were more likely to see connections between their supervisors and who they wanted to become, and (2) incompatibility in candidates’ identities and those of their supervisors and the profession more generally interfered with their ability to construct a future professional identity.

#### Identity Connections

We observed that students’ identity resources were associated with the kind of connection they reported having with their supervisors. In particular, looking across interviewees’ responses to the questions of whether their supervisor represented their profession, helped them to know how to get ahead and was a personal role model, three major ways of relating to the supervisor emerged (Table 4 provides an overview of these themes): (1) as a *Doing model*, which described a supervisor as having particular skills and behaviors; (2) as a *Guidance model*, which described a supervisor able to provide personal guidance and support; and (3) as a *Being model*, which described a supervisor as someone who embodied a future self, or desired aspects of a future self.

**Table 4:** Illustrative quotes relating to different forms of identity construction.

Illustrative quotes
**Theme 1: *Doing* model**
I06: Someone we can observe and learn.
I09: Wouldn’t use role model for workplace, would use mentor. A mentor sets examples and tells you how you should be doing things.
I03: this person is a genuinely good person. (Pause) Doing good stuff and do no harm …profession and personal life
**Theme 3: *Guidance* model**
I05: Someone you can look up to. Someone who can be a guiding hand. Someone who can tell you when you are about to make a mistake and how to fix it.
I13: Someone who lives by their word and who shares those values. They then provide a road map for you to follow.
I21: Um, I think it’s their responsibility to be like you know almost like a guide and like they have to, you know they have a lot of experience, they’ve done this, um, for long time. Um, they’re the ones that have like (.) um you know, the knowledge and they’ve done a Ph.D. before so they know exactly what you know, it’s all about. I think they, it’s their responsibility to guide you through the process and like look out for things that you may not necessarily be aware of and like, point them out to you, um and to you know, um, be, be a link.
**Theme 2: *Being* model**
I04: I guess someone that you look up to, that you kind of think is a bit impressive in their respective field, or their personal life, or something that’s just a bit like, yeah I want to be like that…role modeling is a bit like patterning…
I02: Someone who does exemplify by their characteristics that I value like things that I want to be like and (.) and further in down the track in a place that I see myself potentially being in the future and then someone who sees me as well.
I07: Role model is something that we can learn about. Person has the qualities that I want to be like.

Importantly, candidates with single student identities tended to describe the *Doing* style of role modeling. For these Ph.D. candidates, their descriptions were impersonal and somewhat passive, containing no reference to themselves as participants in the modeling process, and only describing supervisors as targets of impartial observation – “[someone who] *sets examples and tells you how you should be doing things*,” [I09, Civil Engineering].

For candidates with multiple academic identities, they articulated supervisors as guiding and supporting them through the Ph.D. process – “*Someone who can be a guiding hand. Someone who can tell you when you are about to make a mistake and how to fix it*” [I05]. However, candidates who defined themselves in terms of multiple mixed identities tended to describe a *Being* model of supervision, articulating how the supervisor represented a future version of the self – “*down the track in a place that I see myself potentially being in the future*” [I02].

The interviews suggest that a wider range of self-aspects as well as higher levels of proactivity in identity construction may explain why multiple identity resources may have facilitated the establishment of a strong connection with their supervisor as an example of who they could be. Specifically, these candidates talked of agentically *selecting* the aspects of their supervisors which they thought were most aligned with their future identity needs. One candidate [I21, F, Materials Engineering] described how she was able to connect different elements of herself to the different yet complementary aspects of her supervisors:

I21: *Um, I think, like I think aspects of both of them. They’re very different. They’re like incredibly different people. Um, and they’re not, and I wouldn’t want to emulate like either one of them in their entirety, um just because I think also think they’re fundamentally different from me. Like, where all three of us are, like entirely different people. But there’s aspects of both of them, um and they’re different aspects that I think (.) act as like, “Okay that part of, that part of me I want to be more like (.) her. And that part of me I want to be more like that, and I think that’s something I would like to emulate.”*

Another candidate explained how she constructed her own identity by “borrowing” different aspects of her supervisors:

I07 [F Communication]: *But other than that, we need to find (.) our (.) own (.) like uh quality that you really want and then we can borrow from this, this, this, this (gesturing borrowing from something) and then something we need to create–it on. Our identity (.)–build our own identity. So, I don’t think that I lack of (laughs) role models around me…Yes. But I can pick up from–from many and some I need to construct myself.*

#### Identity Incompatibility

In line with a social identity theorizing, we observed that candidates’ perceptions that there were important incompatibilities between their identities and those of their supervisors could present a barrier. In particular, an experience of identity discontinuity (e.g., in the form of, gender or cultural difference) between the candidate and their supervisor – as representative of a future academic identity, could inhibit this process. What follows presents a sample of some of the issues of identity dissonance evident in our sample. It is worth noting that whether these potential disablers of the identity construction process had negative effects was multiply determined, and factors such as the overall set-up of the supervisory team as well as a Ph.D. candidate’s experiences of her or his overall learning environment could act as buffers.

Barriers to the identity construction process were often reported to result from differences related to gender, culture, value or experience. For example, a female engineering candidate described her struggle with the limited range of gender identities within academia:

I01: *The women I know are in the academia they are um (pause)– a lot of them turn– turn to like a more of a male um personality. They become very harsh and dominant. And–And in order to like sort of um be successful you have to turn into– and I was going that way when I was a TA. I turned myself in to this rough, like very (.) um strong woman uh with the same (.) words that a male would have and this– that’s not me.*

This particular candidate went on to talk about the outcomes of this sense of identity disconnection:

I01: *All of that is hidden and it’s not part of– and I’ve– and we’re not one– we’re not one person at work and one person– we’re a whole person and we should be able to bring that uh naturally. We don’t– we should be ourselves and that’s not happening in academia specifically in the– in the engineering.*

Speaking to the issue of cultural identity discontinuity, one female international candidate described the beginning of her Ph.D. in Australia as an “*existential crisis*,” [I17, F, Health and Social Science] that was embodied in her “*fraught*” relationship with her supervisors. She described not fully understanding Australian ways of being and she used the example of not knowing how to appear in different social situations, such as an interview… “*So, even things that like (.) like I said, I’m not even aware of (.) might be important.*” This candidate described how, at the start of her Ph.D. she tried to be “*more personable*” with her supervisors (“…*because I thought that was what Australian lecturers were like*”) but that didn’t work for her, and so she then tried to keep “*things strictly professional.*” This is seen in the following extract, in which she reflected on the way in which cultural differences are experienced in day-to-day interactions:

I17: *And even more importantly I don’t have a strong network within the school because, I mean, like it or not, it’s an all-boys or girls club. So [pause] it’s not just international students or Australian students or whatever, but it’s really how you connect with your faculty other than your supervisors. And [supervisor] “D” was very clear about this. He said, you know like, like, someone asked him, another student asked him, (.) “how do people get grants?” and (.) he joked and he said “well basically I just open my door and walk down my corridor and whoever happens to be there [laughs] gets named on the grant.” So, I don’t appear at school (-) and most Ph.D. students don’t appear at school very often. So, we don’t have that network, we don’t have that rapport. And I think it’s also cultural, because I (.) I don’t see them, I don’t see other academics as peers, I see them as authority figures. I see them as teachers. So, I’m not about to (.) like slap some guy on the back and be like “hey how have you been? How was your day?” It, it’s just not me. You know I, I maintain a, a, a, a healthy professional working relationship with my sup- all my supervisors.*

## Discussion

In this paper, we aimed to understand how Ph.D. candidates went about constructing a future professional identity, and the role of multiple identities and identity compatibility in their construction attempts. While there are a growing number of students around the world who are taking advantage of the opportunity to undertake a Ph.D., the number of academic jobs on offer to them has largely stagnated (Mangematin, 2000). Of particular concern, women and members of minority groups are less likely to feature among the fortunate few who successfully transition from their Ph.D. to academia (Anders, 2004; Robinson-Neal, 2009).

To shed light on the processes of identity construction and its role in unequal professional outcomes, we drew on the SIMIC, which argues that the process of identity change is affected by having access to multiple identities. Although the identity construction context differs in important ways from those typically examined by SIMIC, our analysis suggests that multiple identities may be an important resource here too. In particular, we found that those Ph.D. candidates who were able to access multiple identities – whether in the form of distinct professional groups or multiple perspectives on their identities within the Ph.D. context – reported greater levels of certainty about who they were, who they wanted to be, and how their Ph.D. was helping them to get there. The benefits of multiple identities appeared to be particularly marked when they crossed professional domains (i.e., academia and another vocation). This could reflect the possibility that multiple mixed identities contribute to a richer and more multi-faceted sense of self; it is also possible that Ph.D. candidates who are able to access non-academic identities are particularly well placed to understand how the Ph.D. contributes to concrete professional goals and outcomes. Consistent with the latter possibility, these Ph.D. candidates were particularly likely to see their Ph.D. as a job, rather than an apprenticeship.

We also found that multiple identities appeared to provide a basis for seeing the Ph.D. supervisor (likely to be the most salient exemplar of the academic profession) as a model of who one could become in the future. There was some suggestion that this was because students with a richer and more multi-faceted sense of self were better able to identify and select aspects of their supervisors that they wished to emulate and become. We also found that Ph.D. candidates’ perceptions that who they were was not compatible with the identities of their supervisor, obstructed their identity construction attempts. These findings align with previous work that claims that role models may have beneficial career outcomes by providing people with a bridge to a future self (e.g., Morgenroth et al., 2015), and extend them by suggesting that multiple identities may help people to find and use role models as they travel along their career paths. They also suggest that to the extent that underrepresented or minority groups have fewer identity resources (e.g., because they have moved countries) or experience greater identity incompatibility with their supervisors (e.g., as women in science) this may contribute to greater difficulties in constructing a future identity that will help them to advance in the profession. In this way, this analysis points to a novel leak in the academic career pipeline.

### Implications

Whilst the experience of identity incompatibility was not rare among our sample, its particular form varied widely from one individual to another. In other words, it was not possible to identify which particular constellation of minority identities would form the basis for identity dissonance, as this was very much manifest in the eye of the beholder (Morgenroth et al., 2015). If this pattern were found to hold among Ph.D. students more generally, it has clear implications for the kinds of interventions that educational institutions should consider if they wish to support identity construction. In particular, the typical role model intervention – which involves wheeling out a highly successful member of a minority group for a brief presentation – is unlikely to work. This is not only because it is difficult to select the person who is most likely to be relevant to the identity incompatibility experienced by Ph.D. candidates, but it is also because the process of modeling needs to function on many levels – what to do, how to get there, and who to be.

Indeed, our research suggests that it is only by embedding students in diverse networks of professions (both inside and outside of universities), and peers, and by encouraging them to build and maintain their important groups both inside and outside of their Ph.D., that universities are most likely to support candidates in their attempts to construct the future career identities that underpin thriving and later success. That is, as universities continue to reshape the Ph.D. from unstructured apprenticeship to a more structured program that targets a range of competencies, they would do well to increase the opportunities for students to accrue more Ph.D.-related identities.

### Limitations

At this point, it is important to acknowledge some major limitations of this work that temper our analysis and, especially, the conclusions that we reach. Our sample was not representative, and as we note earlier, had an over-representation of women, and in all likelihood, international students. It appears that those who are likely to experience identity incompatibility (under-represented groups) may have been more likely to self-select into the study, and to tell their story. Indeed, many of the participants in the course of their interview expressed a desire to tell their story in order to help other Ph.D. students who may find their experience a challenging one.

## Conclusion

The importance of this research is two-fold. First, by investigating the mechanics of identity construction, we can begin to better define and so understand the outcomes of this process, particularly with a view to understanding continued inequalities in professional outcomes. Second, once a social identity model of identity construction has been fully developed and tested, we can use this knowledge within applied settings to implement more effective processes and procedures to ensure that any barriers to the identity construction process are not only articulated but also addressed.

## Author Contributions

SB wrote the manuscript and conducted the main thematic analysis. KP, SH, and KG provided editorial guidance. KP provided an independent secondary level of analysis. SB and KP performed the analytic process and collaborated with SH and KG on the analytical findings.

## Conflict of Interest Statement

The authors declare that the research was conducted in the absence of any commercial or financial relationships that could be construed as a potential conflict of interest.

## Footnotes

1 We found no evidence that the emergent themes had any relationship to the year of Ph.D.

## Appendix

### Interview Transcription

The constructs addressed in this paper are (a) interviewees’ perspectives on their own identities in the context of their Ph.D., (b) their thoughts on the proto-typicality of their chosen identity within the academic context, (c) the compatibility of that identity with other identities, and (d) their supervisor’s identity. The data relating to these constructs was drawn from various sections of the interview, and these sections are outlined below, and highlighted in yellow.

### Interview Schedule for Ph.D. Students

Please note that the schedule below does not need to be read out verbatim, but should serve as a guide and prompt to your own conversational style.

You do not need to ask every single question if you feel that some of the topics have already been covered during the conversation*’*s flow. Take notes to help you keep track.

Required:

1. Interview schedule
2. Information sheet
3. Consent form
4. Reflection notes form
5. ipad for recording
6. Pen to take notes

### Introduction

Hi, I’m [name], a student in the School of Psychology, and I am working with Sarah Bentley and Kim Peters on a project that aims to explore people’s understandings of academic professions and the Ph.D. supervision relationship. This interview will take about 50 minutes, and with your permission I will use my ipad to audio-record it for later transcription.

Here is the information sheet, which will provide you with further information about this study.

Please read this through carefully, and sign the consent form if you are happy to take part today.

[Hand over information and consent sheets, and ask the interviewee to complete them. You will then need to sign the consent form as the witness].

Do you have any questions before we start?

### Background Information

To start with, I’d like to find out a little more about you and your supervisory relationships.

1. So what discipline do you work in? What year of you Ph.D. are you in, and what milestones have passed?

[The next question is to just get things warmed up, and to encourage the interviewee to talk generally about why they are doing what they are doing. Try and be as minimal as possible with these prompts to allow the interviewee to talk freely rather than you steering them]

1. So why are you doing a Ph.D.?
2. … and what does it mean to you?
3. Do you see the Ph.D. a job? An apprenticeship? Or are you trying to answer a specific research question?
4. Can you tell me how and why your research question came about?
5. Tell me about your supervisory relationships…
  a. What is the supervisory set-up (number of supervisors, supervisory ratio, e.g., 50/50, 20/80 etc.)
  b. How did you choose your supervisor/s?

I’d like to hear a little about the day-to-day structure of your supervisory set up:

a. How often do you meet?
b. With whom (i.e., one supervisor or all both, in the case of multiple supervision)?
c. On a regularized or *ad hoc* basis?
d. Who manages the agenda, and who leads the meetings?
e. Are there lab group meetings, and if so do you go to them?
f. Do you have interactions with other Ph.D. students?

### Your Professional Identity

Now I’m interested in finding out about your identity or sense of who you are at work. People who work in universities use various different terms to describe their profession or role, including researcher, academic, scientist, economist, geographer, student and so on

[(a): Interviewees’ perspectives on their own identities in the context of their Ph.D.]

When you meet new people and they ask what you do, what do you tell them?

1. Is this the best way of describing what your profession or work is about? If not, what term would be more appropriate?
2. Looking back, when did you start to think of yourself as being a [interviewee*’*s term/s]? Five years ago did you think this is what you would be doing? Is it how you imagined it would be?
3. Can you see yourself continuing to be a [interviewee*’*s term/s] in the future? What impact, if any, will completing your Ph.D. have? If you think your profession will change, what will it be then?

[(b) their thoughts on the proto-typicality of their chosen identity within the academic context]

1. Can you tell me what it means to be a [interviewee*’*s term/s]. That is, what are the goals, values, characteristics and behaviors that you associate with the ideal [interviewee*’*s term/s]?
2. Looking back over your career^∗^ so far [^∗^for a younger interviewee this maybe be “educational pathway” rather than career], how has your understanding of what it means to be a [interviewee*’*s term] changed?
3. What has affected your understanding of what it means to be a [interviewee*’*s term]?

[If the interviewee is finding it hard to articulate any of the above, try to find out why this is: “Do other Ph.D. students also find it hard to identify their profession and its goals and values?”]

[(c) the compatibility of that identity with other identities]

1. Do the goals and values that you mentioned before match your own personal goals, values and characteristics?
2. Do you feel you fit in in [interviewee*’*s term/s]?
3. Do you feel that being a [interviewee*’*s term/s] is compatible with the rest of your life and the people in it? Does it fit with how you see yourself as a person?

### Your Profession and Your Supervisor

*In this section I am going to you a few questions about your own Ph.D. supervisor. In the first instance, I’m interested in your thoughts about the relationship between your supervisor and your identity as a [interviewee’s term]*.

[Remember, if there are multiple supervisors, adopt the questions below to cater for this]

[(d) their supervisor’s identity]

1. Do you think your supervisor is a typical [interviewee*’*s term/s]? That is, does your supervisor exemplify the values, characteristics and behaviors of the ideal [interviewee*’*s term] that you mentioned earlier?
  a. If yes, in what ways? If not, why not?
2. Does your supervisor help you understand what you need to do to advance in your career in the way you want to? [For those students who are looking for a career outside of academia, does their supervisor know this, and has the supervisor encouraged them to consider academia?]

I am going to shift the focus of the interview a bit now to ask about role modeling. However, before we talk about your experiences of role modes, I want to find out what this term means to you in a very general sense.

[Identity navigation]

1. What do you think a role model is? If you were to say that someone was a personal role model for you, what would you mean?
2. On the basis of that definition, would you say that your Ph.D. supervisor is a role model for you?
  a. If yes, why? If no, why not?
3. Is there anyone else who is a role model for you currently in your work?
  a. If yes, who is this person and why are they a role model?
4. Do you ever feel that you lack role models in any aspects of your work?
  a. If yes, what were you looking for?
5. You said that your supervisor [is/is not] a role model for you. Do you think that this matters in anyway for your Ph.D. and career development? In other words, would anything be different if you had a supervisor who [was not/was] a role model for you?

### The Supervisory Dynamic

In this final part of the interview, I am interested in your thoughts about the importance of the Ph.D. supervisory relationship and the different responsibilities of students and supervisors.

[Reflection on Identity navigation]

1. Research suggests that the Ph.D. supervisor/student relationship is of central importance when it comes to the learning and career outcomes of the student.
  a. Why do you think this is?
  b. How does this map onto your own experience?
2. Thinking about your experience as a Ph.D. student, do you think that a supervisor is responsible for the success or failure of their students?
3. What *specifically* do you think a supervisor is responsible for in the context of the relationship, and what is the responsibility of the student?

[If not mentioned, you can ask about responsibility for learning, teaching, designing research questions, getting publishable findings, showing initiative, passion, keeping on schedule etc.]

1. In what specific ways does your supervisor meet/not meet his or her responsibilities as a supervisor?
2. If you were supervising a student, would you do things differently?
3. Do you think you are a good Ph.D. student?
4. …why do you think that? (For instance feedback from supervisor? Given or sought?)
5. Do you think your supervisor/s thinks the same?
6. Do you envisage working with your supervisor beyond your Ph.D.?

Thank you so much for your time.

In our research we are interested in in the different kinds of supervisory relationships that occur in different disciplines and how they impact on the success or otherwise of the Ph.D. student’s career. Is there anything else you would like to talk about with regard to this question?
